# Close-Range Coordination to Enhance Constant Distance Spacing Policies in Oversaturated Traffic Systems

**DOI:** 10.3390/s24154865

**Published:** 2024-07-26

**Authors:** Kay Massow, Niko Pfeifer, Fabian Ketzler, Ilja Radusch

**Affiliations:** 1 Fraunhofer Institute for Open Communication Systems (FOKUS), 10589 Berlin, Germany; niko.pfeifer@fokus.fraunhofer.de; 2 Daimler Center for Automotive IT Innovations, Technische Universität Berlin, 10587 Berlin, Germany; fabian.ketzler@dcaiti.com (F.K.); ilja.radusch@dcaiti.com (I.R.)

**Keywords:** CACC, constant distance spacing, traffic light, signalized intersection, vehicle simulation, traffic simulation

## Abstract

In the pursuit of string stability within CACC (cooperative adaptive cruise control) platoons, prevalent research has favored constant time gap (CTG) spacing policies; namely, vehicle interspacing increases linearly with the speed. Although constant distance gap (CDG) spacing policies have greater potential to enhance traffic capacity, they suffer from notable limitations regarding string stability and diminished safety margins at high velocities. In our previous work, we proposed applying CDG in specific scenarios, such as starting platoons at signalized intersections, where traffic throughput is critical and safety requirements can be met due to relatively low speeds. We demonstrated the substantial potential of CDG to increase the capacity of signalized intersections under oversaturated conditions. However, our study also revealed potential performance drops of CDG in dense traffic networks. To address these issues, we propose close-range coordination between vehicles to (1) limit platoon length, (2) create gaps for merging, and (3) avoid entering intersections when there is a high likelihood of stopping within the intersection area. In this paper, we extend our previous work by implementing these three measures. We successfully evaluate their positive impact on CDG’s performance in entire traffic systems through large-scale traffic simulations involving several thousand vehicles, thereby affirming our earlier hypothesis

## 1. Introduction

Cooperative adaptive cruise control (CACC) systems enhance traffic efficiency and safety as an automated way to enable vehicles to form platoons and maintain close distances while traveling. CACC integrates wireless communication with onboard sensors, allowing vehicles to follow those in front of them more accurately, respond faster, and maintain shorter gaps. This approach optimizes traffic flow, improves safety, and reduces fuel consumption and emissions by reducing air drag and implementing smoother acceleration profiles [1,2,3,4,5,6,7,8].

Recent interest in CACC has focused on spacing policies to optimize traffic flow within platoons. In our previous study [1], we explored the efficacy of constant distance gap (CDG) spacing policies in specific driving scenarios, notably emphasizing their potential to improve traffic capacity, particularly at signalized intersections. Our underlying hypothesis is to employ CDG selectively in specific driving scenarios, aiming to maximize its benefits through a context-aware switch between constant time gap (CTG) and CDG. This switching depends on the simultaneous occurrence of the following conditions:

1. Traffic throughput is of crucial importance;
2. Platoon sizes are short enough that communication topology complexity and string instability can be handled, e.g., employing a mini-platoon control strategy [2];
3. Smooth and predictable accelerations at low velocities cover safety requirements.

Among several use cases in which such conditions prevail, clearly, traffic light-controlled intersections are one of the most relevant. At oversaturated, traffic light-controlled intersections, traffic throughput is of crucial importance as they are the bottlenecks in traffic. At intersections, the traffic flows of two crossing streets share one spot in a time-duplex manner. Platoons start at the traffic lights with low velocity and the platoon sizes are inevitably limited due to the signal phases cutting platoons.

While our study [1] demonstrated the promising capacity enhancement capabilities of CDG at intersections, it also revealed limitations that hinder seamless integration into broader traffic systems. These limitations include issues arising when CDG is deployed at high penetration rates in very dense and oversaturated traffic conditions. Specifically, the challenges are related to prevented lane changes, blocked intersections, and insufficient coordination among vehicles within CDG platoons under such conditions. Building upon these findings, this paper aims to address the named limitations through three measures:

1. **Platoon length limitation (PLL):** Implementing mechanisms to control the length of platoons to mitigate potential congestion and facilitate smoother traffic flow.
2. **Intersection awareness (IA):** Preventing vehicles from entering intersections when stopping within the intersection is likely, thereby avoiding blockages.
3. **Creating merging gaps (CMG):** Creating gaps within platoons to allow for merging and lane changes, improving coordination.

This study involves comprehensive large-scale traffic simulations involving several thousand vehicles to evaluate the effectiveness of these measures in enhancing CDG policies within broader traffic systems. The study maintains the boundary conditions from our original research [1], focusing on traffic light-controlled intersections and urban speeds up to 50 km/h. A one-vehicle-lookahead communication pattern is employed to keep the communication topology realizable in dense traffic, i.e., as simple as possible [3]. String stability is assumed to be achieved either by a limited platoon length or falling back to the mini-platoon communication pattern [2], which exhibits the lowest possible communication overhead compared to the one-vehicle-lookahead pattern.

The research questions discussed in the rest of this paper focus on capacity improvement of CDG over CTG at signalized intersections. We explore how PLL, IA, and CMG impact this improvement, extending our original study [1] while keeping the experimental setup and simulation models consistent. While the original study [1] was focused on investigating the fundamental benefits of CDG and on exploring the practical implications of the tested CDG spacing policies for enhancing CACC in real-world scenarios, the present study focuses on implementing the mentioned measures to address the drawbacks revealed in the original study. To avoid repetition, we refer to the original study for detailed considerations.

After discussing related work in Section 2, the rest of this paper is structured as follows. Section 3 outlines the methodology of our study, the fundamental idea behind its structure, the simulators, and the models we use. In Section 4, we present our method used to transfer the precision of simulating a CACC controller in a vehicle dynamics simulator to the traffic simulator level. The extensions to the models of our original study are described in Section 5. In Section 6, we present the results of the extended study and discuss their findings in Section 7. We conclude this paper in Section 8.

## 2. Related Work

In this section, we review existing concepts concerning the extensions introduced in this study compared to the original study [1]. This work contributes to the literature by improving the capacity of CDG over CTG at signalized intersections, incorporating the three measures of PLL, IA, and CMG. Specifically, we focus on (1) limiting platoon length, (2) creating gaps for merging, and (3) preventing entry into intersections when a stop within the intersection is anticipated. We limit the review of related work to these three elements to avoid repetition of the work in [1].

Understanding the significance of these measures requires familiarity with the research landscape within the platooning domain. For comprehensive surveys on platoon coordination and spacing strategies, refer to [1,4] respectively.

### 2.1. Limiting the Platoon Length

The performance of CDG in multi-intersection scenarios is significantly affected by the ratio of intersection interspace to platoon length [1], which is indirectly regulated by the duration of green-light phases. Thus, the platoon length should be constrained, ideally considering the intersection interspaces and the traffic light configuration [1] in addition to stability and safety aspects in general.

For limiting the platoon length in this study, we consider several aspects. Firstly, we need a reference value for the maximum platoon length in general and tailored to multi-intersection scenarios. Secondly, given the substantial volume of vehicles in the scenarios under consideration, the method for constraining the platoon length should be straightforward and decentralized, operating without communication overhead and without the need for a formal platoon architecture.

As a general guideline for platoon lengths based on stability considerations, Ref. [5] suggests an upper limit of 20 vehicles for CTG. A literature review did not yield specific findings favoring shorter platoon lengths in multi-intersection scenarios. Ref. [6] considers shorter platoons but indicates that longer platoons yield better performance.

In centralized platoon management approaches [7], the platoon leader establishes links with each subsequent vehicle, enabling storage of all relevant platoon parameters, including current and maximum length. However, in decentralized platooning, an information exchange along the selected communication topology is required to determine and limit the number of vehicles within the platoon. For our preferred one-vehicle-lookahead topology, we draw inspiration from the approach in [7] in shaping our strategy for constraining platoon size. Beacon messages include Platoon ID and Platoon Depth fields. Platoon ID serves as a distinct identifier for different platoons, while Platoon Depth, indicates the vehicle’s position within the platoon. The platoon leader holds a depth value of 0, with increasing values indicating subsequent positions within the platoon.

### 2.2. Creating Gaps for Merging

To facilitate the creation of gaps for merging of vehicles into platoons, there is a distinction, as previously discussed, between two approaches: the centralized approach, wherein the leader handles platoon management; and the decentralized approach, wherein each member vehicle holds platoon information and determines its behavior.

Most platoon merging approaches in the literature follow the centralized approach, for example, the work in [8], developed for the Grand Cooperative Driving Challenge 2016, heavily relies on V2X messages and depends on the platoon leader to coordinate the platoon.

In line with our preferred decentralized approach, Ref. [9] builds upon the concept of virtual platoon leaders, a role that can be adopted by each vehicle within the platoon. Moreover, Ref. [10] proposes a protocol managing multiple join and leave maneuvers without relying on a platoon leader, yet necessitates communication with the roadside infrastructure. Conversely, the method outlined in [11] enables individual vehicles to autonomously handle platoon information but requires bidirectional communication.

In meeting our requirement for unidirectional communication, our gap-opening strategy not only requires a decentralized approach but also must function for human-driven vehicles without communication. Considering potentially high, yet not 100%, penetration rates, traffic must operate alongside non-connected vehicles. While Refs. [12,13] address mixed traffic scenarios at signalized intersections, they assume wireless communication for all vehicles and consider a formal platoon architecture.

Ref. [14] introduced a decentralized platooning merge-gap creation method inspired by biological systems, such as ants, to address the limitations of decentralized platooning. This approach relies on a relatively extensive set of rules. The rule set of the approach still requires communication among all vehicles, as well as mutual localization. Consequently, we do not adopt this approach directly; however, we aim to develop an approach inspired by it, utilizing a single rule and operating without wireless communication.

### 2.3. Intersection Awarness

Assuming a traffic backlog from a traffic light reaches an adjacent intersection, vehicles may stop in the middle of the intersection and remain there until the light changes for cross traffic. This situation, subsequently referred to as junction blocking [1], forces cross traffic to wait for a full light cycle before the intersection clears. Due to the close distances in CDG platoons, this issue occurs more frequently than with CTG, which naturally causes the platoon to contract while stopping. Even when vehicles stop to avoid junction blocking, traffic backlogs can prevent turning, leading to turn blocking [1], where cross traffic behind the turning vehicle is blocked for the current light cycle. Our goal in this work is to enable vehicles to anticipate unintended stops within intersection areas.

Refs. [1,15] delve deeper into issues arising from intersection blocking. While many studies address junction blocking by attempting to mitigate the problem through traffic signal control or by cooperation of vehicles with traffic lights for traffic coordination, these approaches are not pertinent to our study and are thus not discussed.

One of the few works attempting to address the issue from the perspective of individual vehicles through foresight is [16]. The no-block heuristic described therein is a part of the intersection model within the traffic simulator SUMO. In our previous study [1], we used SUMO version 0.32, where the no-block heuristic malfunctioned for sub-second simulations (our CDG model for SUMO requires a step size of 0.1 s). These issues were resolved in SUMO version 1.6. Therefore, in this work, we can now utilize the improved version of the SUMO heuristic and propose a method for implementing this heuristic in a similar decentralized manner in the real world.

## 3. Methodology

In this section, we outline the methodology employed in our study. The underlying concept behind its structure is as follows. Assessing the impact of starting CDG platoons at signalized intersections on traffic flow requires accurately representing control dynamics and considering the broader traffic network at the same time. This approach aims to capture small-scale control effects that have significant impacts on overall traffic patterns.

The study builds upon the structure of our original work [1] and expands to include three additional measures: PLL, IA, and CMG. We begin by describing our modeling of CACC platoons, the various spacing policies under investigation, simulation models for different scenarios, and the evaluation metrics used.

### 3.1. Analysis Approach

Developing and evaluating control systems like CACC through simulation requires accurately mapping vehicle dynamics. Small variations in how physics and control systems interact with the environment can lead to significant differences in the resulting behavior, especially when evaluating impacts on entire traffic systems.

In order to assess the intersection capacity improvement of CDG over CTG for starting platoons at oversaturated, signalized intersections, it is not sufficient to consider one platoon at an isolated traffic light. On the contrary, to assess the impact of CDG on multiple mutually influencing intersections we need to include a plethora of permutations of the signalization, while being physically realistic at the same time.

**Figure 1:**
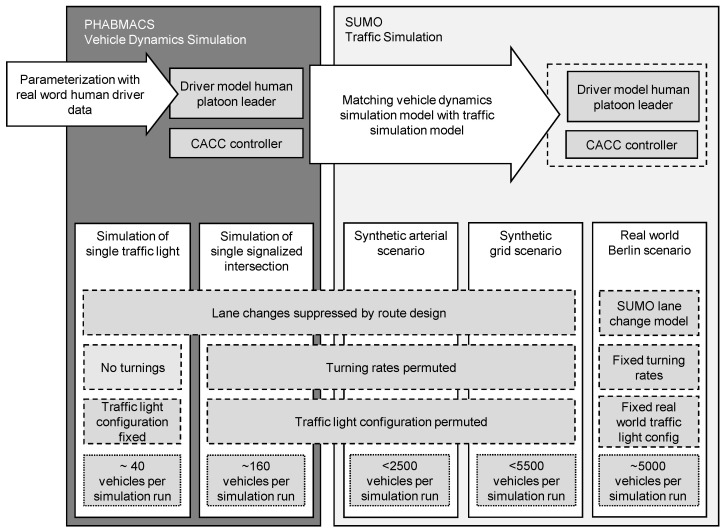
Analysis approach of the study, summarizing the used simulators, scenarios, simulation models, and parameterization.

As we have contradicting requirements in this regard, our study employs different kinds of simulators and builds systematically from studies of a single isolated traffic light, to an intersection, a synthetic corridor and grid network, and a real-world corridor, providing a logical sequence of increasing complexity. The term single isolated traffic light refers to a traffic signal positioned on a straight road, often resembling a pedestrian light in its singular and standalone control function. The different parts of the study are depicted in Figure 1, summarizing the used simulators, scenarios, simulation models, and parameterization. For studying CDG at a single isolated traffic light, the sub-microscopic vehicle simulator PHABMACS [17] is the appropriate tool. Thanks to its ability to scale out physics and control algorithms, simulating a whole intersection including hundreds of vehicles for hundreds of simulation runs is enabled [17].

To research entire traffic systems involving thousands of vehicles, PHABMACS becomes out of scope. Therefore, we match the implementation of CACC controllers in PHABMACS and its validated vehicle model to the SUMO [18] traffic simulator. Our method for calibration and validation ensures that the traffic simulation model in SUMO generates the same results regarding relevant metrics (see Section 3.5) as the vehicle dynamics simulation model in PHABMACS. This step, briefly covered in our original study [1], is detailed further in Section 4.

In the macroscopic analysis, we begin the examination of an entire traffic system using two synthetic scenarios to reveal the relationship between CDG and specific configurations of road topology and traffic light settings. Vehicle routes in these synthetic scenarios are designed to eliminate lane changes, isolating their potential impact. We permute the configurations of traffic lights and turning ratios to highlight edge cases. Subsequently, in a real-world scenario, we evaluate the performance of an actual area within a traffic system. The road layout and traffic light configuration for this real-world scenario are derived from real data and we assume uncoordinated lane changes. For all simulations, we saturate the inlets of the scenario with the maximum possible traffic flows in order to create an oversaturated condition. This enables us to measure the capacity of the intersections, which is defined as the maximum possible traffic flow.

The extensions made to the original study [1] pertain solely to the simulation scenarios at the traffic system level using SUMO. These modifications address effects occurring solely within entire traffic systems, rather than individual intersections. Consequently, the simulations conducted in PHABMACS are not replicated or analyzed here; only the simulations performed using SUMO are considered.

### 3.2. Modeling of the CACC Platoon

**Figure 2:**
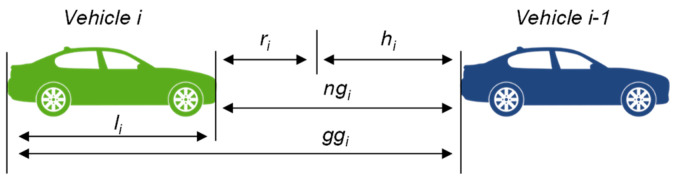
The formal platoon model used in this study.

In the following, we introduce our platoon model and its relevant parameters. The relevant relationship between throughput and platoons passing the traffic light is the number of vehicles per platoon length. The portion of platoon length pertaining to each vehicle in a CTG platoon depends on the parameters depicted in Figure 2. The constant portion is the *i*th vehicle length $l_{i}$ plus the standstill distance $r_{i}$, while the dynamic portion is the time gap $h_{i}$ (${ng}_{i}$ is the net gap and ${gg}_{i}$ is the gross gap), which grows with the platoon velocity. The dynamic part is zero in CDG platoons, i.e., the CDG platoon length is always the same as in standstill, which is what makes the CDG so effective.

We assume fully automated longitudinal control for all vehicles in the platoon with no driver in the loop, as required when driving with very small inter-vehicle gaps. To achieve comparability of different spacing polices, we neglect the driver’s reaction time for all considered spacing policies. This consideration is especially relevant for the start-up at traffic lights, as human reaction time would make a notable difference here.

The acceleration profile of the platoon leader to the target speed (50 km/h within urban areas) significantly influences the time in which the platoon crosses the intersection and the associated potential throughput improvement of CDG over CTG. To ensure realism in our study, we decided to adopt an acceleration profile for the platoon leader based on real-world data. This profile is derived from average human driver behavior acquired from 3546 start-up situations in a field experiment involving 98 human drivers (73 male, 25 female), as obtained in [1].

### 3.3. Spacing Policies and Parameterization

As mentioned earlier, CDG should not be applied at arbitrary high velocities due to safety aspects and stability issues arising when the one-vehicle-lookahead communication pattern is applied. Thus, there is a speed limit at which the CDG spacing policy is required to be switched to CTG. For the threshold of this speed limit we chose 50 km/h and 30 km/h as parameters to be studied in the simulation, as motivated by [1]. This policy is referred to as SWITCH in the remainder of this work.

To compare CDG with CTG, the constant portions ($l_{i}$ and $r_{i}$) need to be parameterized with the same values. For comparison, these values and the time gap of CTG should be chosen to be as realistic as possible, as their ratio makes a considerable difference. Thus, we decided to derive the final parameterization from real-world data: $l_{i}$ = 5.15 m, $r_{i}$ = 2.95 m, $h_{i}$ = 0.87 s. Indications for all these parameters and the detailed method of their derivation are described in [1]. SWITCH is realized as a simple change between *CDG* and *CTG* at 30 km/h, with SWITCH1 defined by ${\;d}_{r,i}=r_{i}+h_{i}{max(0,v}_{i}{-v}_{lim})$, where $d_{r,i}$ is the desired spacing and $v_{lim}$ is the threshold of 30 km/h. SWITCH2 is parameterized with a larger time gap, so that it reaches the same inter-vehicle distance as CTG at 50 km/h. Table 1 summarizes all policies studied in this work.

To facilitate the control of the platoon length and gap opening, discussed in Section 5, $k_{i}$ represents the position of a vehicle i in the platoon, and can be subjected to thresholding by $k_{tr}$. Gap opening in the platoon is achieved by ${tv}_{i}$, representing the time span during which a neighboring vehicle remains in the field of view of i, and ${tv}_{tr}$, which sets a threshold for this time.

### 3.4. Simulation Models

The simulation models used in this study are presented in the following. This includes modeling of the intersection and the simulation scenarios with their parameterization, as well as a controller for the longitudinal acceleration of the vehicles in the platoon.

#### 3.4.1. Intersection Model

The excellent performance of CDG at a single isolated traffic light, as detailed in [1], is largely due to the fact that platoons can pass isolated traffic lights in a free flow. For a comprehensive evaluation of intersection performance, it is important to consider factors that reduce traffic flow, such as slower speeds during turns, stops when yielding, and the limited duration of green lights.

Urban intersection layouts encompass numerous combinations of elements, each of which can have a distinct impact on the performance of CDG [19,20]. As we have to permute many parameters apart from the layout, we define a reference layout that covers as many layout-related aspects as possible and can be a fixed parameter for further studies.

**Figure 3:**
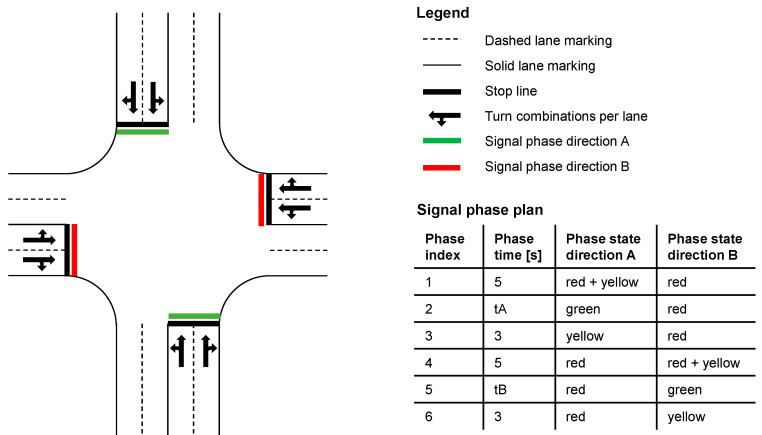
Four-way, two-lane reference intersection layout for simulation.

Figure 3 depicts our reference layout with two lanes in each direction. Each right lane mixes straight driving with protected right-turning vehicles, as there are no pedestrians. The durations for the yellow phases and clearance intervals are fixed. The parameters tA and tB listed in the signal phase plan indicate the green phases for both directions of the intersections A and B, which are varied for the different simulation runs subsequently. Each left lane mixes straight driving with unprotected left-turning vehicles, which always need to wait for oncoming vehicles. This is ensured as the intersection is oversaturated according to the scope of this study. For further details on parameters like the intersection radius, turning speed, and the considerations that lead to this particular intersection layout, the reader is referred to [1].

#### 3.4.2. Multi-Intersection Scenarios

**Figure 4:**
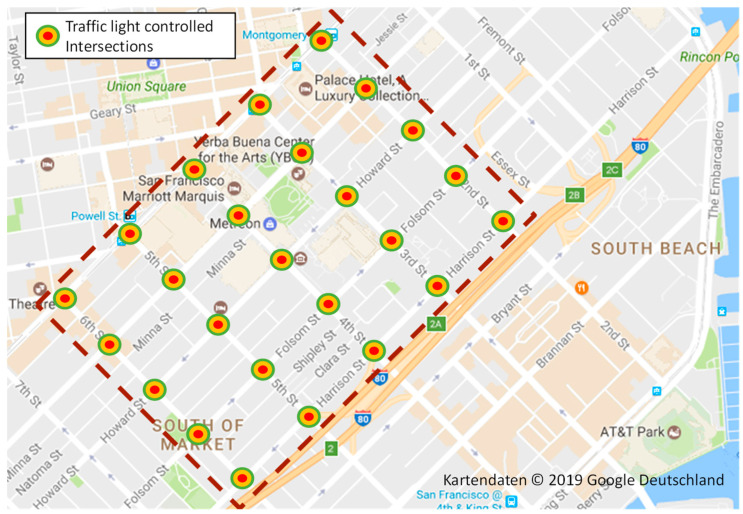
The two synthetic simulation scenarios, arterial and grid, combine the intersection layout of the previous sections with two-way streets (one lane per direction) along the dimensions of an urban area in San Francisco, CA, USA.

In addition to factors that reduce CDG performance at signalized intersections compared to isolated traffic lights, further considerations arise in scenarios with multiple mutually influencing intersections. These include congested intersection outlets affecting off-flowing traffic and reduced in-flowing traffic. To address this very important aspect, we analyze two synthetic simulation scenarios: an arterial corridor [21] with five intersections and a coordinated grid network [21] of 25 intersections, each with the layout and the signal phase plan depicted in Figure 3. The intersections are aligned on a grid with specific distances (276.5 m NW and 192.5 m SE), originating from the area depicted in Figure 4, unified for the simulation with two-way streets. This area serves as a reference point for the geometry of a real-world traffic grid. Specifically, this is relevant for the distances between intersections, a crucial parameter in the context of traffic signal control [1].

In such multi-intersection scenarios, the performance of CDG and CTG is influenced by many different effects, whose impact can be observed as a superposition in the measured metrics. Hence, our objective is to maximize the isolation of as many of these effects through the design of synthetic scenarios, thereby facilitating the interpretation of the results, as explained below. Both scenarios are simulated with multiple permutations of traffic light phases and turning ratios, including up to 5500 vehicles simultaneously:

- Equal intersection interspaces enable isolating the impact of different interspace lengths on the simulation results from the other simulation parameters;
- Vehicle routes are designed to eliminate lane changes in these synthetic scenarios to isolate their impact on the outcomes;
- Oversaturated traffic inflows and unobstructed outflows exclude the effect of fluctuating inflows that hinder the achievable increase in throughput.

**Figure 5:**
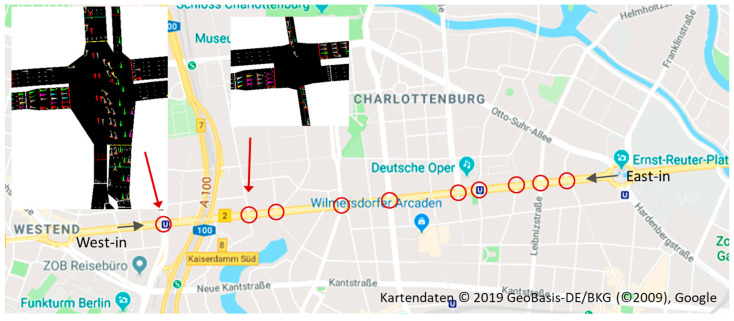
Real-world scenario: arterial road with ten intersections in Berlin (intersections marked by red circles)

In addition to the synthetic simulation, which helps to reveal the relationship of specific arrangements of road topology and traffic light configuration, we use a real-world road network scenario to demonstrates the performance of CDG in a real-world traffic system that mixes a plethora of such arrangements at the same time. This scenario covers a heavily frequented arterial road in Berlin, Germany, depicted in Figure 5, with ten traffic light-coordinated different intersection layouts spaced between 160 m and 500 m apart. The traffic light configurations are fixed and captured from real data (see [1] for the details). We, again, assume a maximum possible traffic inflow and an unobstructed outflow; however, now we include uncoordinated lane changing in the simulation.

#### 3.4.3. Realization of the Controller

The controller employed for the physically realistic simulation part of our study in PHABMACS (refer to [1]) has been developed and implemented for use in real test vehicles. As these simulations in PHABMACS are not conducted anew within the scope of this study, we do not delve into the specifics of the controller, but refer to its description in [1]. To represent this controller in large-scale simulations in SUMO, we employ a simplified model described in the following. This model transfers the fundamental characteristics of the controller to SUMO and is further calibrated and validated in Section 4.

In order to map CACC in SUMO, we choose the Krauß car-following model [22] as the basis implementation. The model is directly applicable for CTG. For CDG, however, we need an adaption of the model, as fixed following distances cannot be realized for the following reason. Although, the Krauß model has a parameter for the velocity-dependent time gap, setting this parameter to 0 s does not make the vehicles start up at the same time. Each vehicle starts exactly one simulation time step later than its predecessor. Since all vehicles follow the same acceleration trajectory, the inter-vehicle distance is constantly growing while accelerating and shrinking while decelerating.

For this reason, we modified the Krauß model [22] for the CDG case according to (1). Similar to the Krauß model, our model is founded on the calculation of a maximum safe speed $v_{safe}$. If the distance s to the predecessor is greater than the standstill distance $s_{0}$, we apply the Krauß model with a small modification. The tolerance band $s_{t}$ is added to $s_{0}$ for the calculation, thereby creating a tolerance band around $s_{0}$. This allows the vehicle to overshoot the stand still distance by $s_{t}$, which is required as a buffer for driving with constant distances. If the distance s is within this tolerance band, $v_{safe}$ is set to the preceding vehicle’s velocity $v_{l}$. If the band is undershot, $v_{safe}$ is set to $v_{l}$ reduced by a factor d (0.95) to make the vehicle return to the tolerance band. The simulation’s step size needs to be aligned with $s_{t}$, in our case $s_{t}$ equals 0.5 m at a simulation step size of 0.1 s.

This is a modification of the SUMO car-following system for certain operating conditions and we would recommend using this model for this specific CDG application only. For our use case, it works sufficiently well, as demonstrated in the next subsection. Another required modification is to enable followers to catch up with the preceding vehicles who drive with maximum speed. For this purpose, we lowered the maximum speed of vehicles without vehicles in front of them to 95% of the speed restriction of the current link in SUMO. This is also performed for the CTG case.

(1) $${\;v}_{safe}=\left\{\begin{array}{cc} v_{l} & s_{0}-s_{t}<s<s_{0}\\ v_{l}d & {s<s}_{0}-s_{t}\\ -b\tau +\sqrt{b^{2}\tau^{2}+v_{l}^{2}2b(s-(s_{0}+s_{t}))} & s>s_{0} \end{array}\right.$$

### 3.5. Metrics

The American HCM [23] and the German HBS [24] define the metrics for signalized intersections based on waiting time, level of service (delay), and waiting queue length in front of traffic lights. Although these metrics should be used as relevant parameter for intersection efficiency, they are not suitable for our study, as we are not aiming for optimization of the configuration of the traffic lights. For comparison of CDG and CTG at intersections, we basically measure the maximum intersection capacity [23] for both. While oversaturating the intersection inlets, we choose to measure the following metrics:

- traffic throughput [1/min]—vehicles passing per time;
- travel time [min]—average time vehicles need to pass;
- traffic density [%]—portion of road meters occupied by vehicles.

The throughput is needed to derive the intersection capacity, while the travel time experienced is a quality-of-service (QoS) measure. We also measure the density to analyze the efficiency of road utilization, which is most relevant when whole traffic systems are considered in the following subsections. Refer to [1] for details of how these metrics were derived.

## 4. Model Calibration for Macroscopic Simulations

The next step for our studies on CDG is to evaluate its impact on whole traffic systems; i.e., on multiple mutually influencing intersections. This step was briefly covered in the original study [1]; therefore, it is elaborated upon in more detail here. As motivated earlier, the development and evaluation of longitudinal control like CACC in simulations requires realistic mapping of physics. Fine differences in mapping physics and the control system interacting with its environment may lead to considerable differences in the resulting behavior. Thus, for studying CDG at a traffic light-controlled intersection, the sub-microscopic vehicle simulator PHABMACS is the appropriate tool (for explanations of the terms microscopic, macroscopic, and sub-microscopic simulation models, see [17] or [22]). For its ability to scale out physics and control algorithms, simulating a whole intersection including hundreds of vehicles for hundreds of simulation runs is enabled [17].

However, in order to research whole traffic systems including many thousands of vehicles, PHABMACS becomes out of scope for two reasons. First, mapping that many vehicles would still require considerable time and computation capacity. Second, traffic systems studied using such a macroscopic perspective may also produce realistic results provided that an appropriate model is leveraged, which maps the microscopic behavior sufficiently on a macroscopic scale.

In the following, we propose our methodology to calibrate and validate a sub-microscopic simulation model against a microscopic simulation model. This method enables the transfer of relevant properties from a sub-microscopic simulation model to a microscopic simulation model without compromising the accuracy of the sub-microscopic simulation model for a specific application case. This allows us to harness the performance of a microscopic simulator to conduct large-scale traffic analyses for this application case involving thousands of vehicles. We use this methodology to match the implementation of CACC controllers in PHABMACS [1] and its validated vehicle model to the SUMO [18,25] traffic simulator. Calibration and validation are essential here in order to ensure that the traffic simulation model (see Section 4) in SUMO generates the same results regarding relevant metrics (see Section 3.5) as the vehicle dynamics simulation model in PHABMACS. We decided to use SUMO for the following considerations. To integrate our vehicle model for CDG, as well as the models for PLL, IA, and CMG into the traffic simulation, we require an open-source simulator (VISSIM is not open source and involves costs). Among open-source simulators, the SUMO simulator has established itself as a validated tool for traffic-related investigations due to its widespread adoption within the research community.

### 4.1. Calibration and Validation Method

Taking inspiration from [26] our proposed validation methodology consists of two steps. First, the models of both simulations, vehicle simulation (PHABMACS) and traffic simulation (SUMO), are calibrated. This calibration aims to match the time and location of each vehicle during the simulation for the same scenario in both simulators. Second, the metrics determined to evaluate the simulation results are determined in both simulators for the same scenario and validated against each other. This model validation method was designed following the consideration of balance between effort and value of model confidence presented in [17]. Accordingly, this method does not aim to find the general validity limits of the model but to assure its validity for a specific context. In our case, this refers to the validity of the model for the considered simulation scenarios and its ability to generate valid metrics for this specific application case.

### 4.2. Step I—Timing Calibration

**Figure 6:**
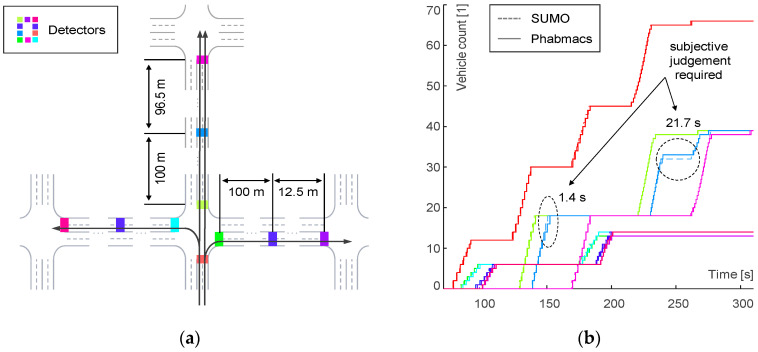
Calibration SUMO-PHABMACS. (**a**) Detector setup. (**b**) Timing diagram: subjective validation criterion. Example: green light 15 s, offset 0 s. Colors match detectors in (**a**).

In order to calibrate the timing and location of a vehicle (time-space domain), we first need to place detectors in both simulations at crucial, scenario-specific locations. In our case, our objective is to consider multiple mutually influencing intersections, i.e., our scenario includes one central intersection and one adjacent intersection in each direction, as depicted in Figure 6a. As the intersection layout is identical from each direction, we just need to regard vehicles coming in from one direction at the central intersection.

The distances between the intersection, as well as the traffic light cycle times and their offset between the intersections, are chosen in accordance with the next section. The detectors are placed according to Figure 6a. Detectors are positioned at both the entrance and exit of the intersection, each located 20 m away from the center of the intersection. Additionally, detectors are placed between the intersections at the distances specified in Figure 6a. The colors assigned to the detectors in Figure 6a correspond to the colors of their respective time graphs in Figure 6b. The solid graphs depict vehicle counts measured in PHABMACS over the simulation time, while the dashed graphs represent similar measurements in SUMO. The time graphs are nearly indistinguishable in the figure because they are almost identical in both simulators. One exception is the blue detector, which shows a different count at the marked circles around times 150 and 250 for more than one second. In this way, the start-up characteristics and travel time through and between intersections are validated. Again, lane changing is disregarded for the aforementioned reasons. Left turns stop the traffic on the left lane and the turning vehicle is the only one passing the traffic light for the current cycle.

To ensure that the timing in both simulations is similar, we run both simulations for all relevant permutations of parameters and compare the vehicle count for all detectors. This process is automated to minimize the manual tuning and validation time. We need to ensure the correct number of vehicles pass per traffic light cycle for all permutations of CDG penetration, traffic light cycle times, and offsets. As the shortest traffic light cycle time to be studied is 5 s, a 1 s maximum difference between corresponding detectors in both simulations is sufficient.

For the assessment of validity, we propose the objective timing criterion, as described above, complemented by a subjective criterion, as motivated by [17], for the following reasons. If a simulation scenario run was invalid and the number of detectors that showed higher differences than 1 s was small, the verdict of validity could be changed manually if reasonable. One example for such a subjective verdict is depicted in Figure 6b. While the objective criterion can be applied automatically, the subjective criterions needs to be assessed manually. The idea here is to apply automatization to the greatest extent, while manually reducing effort to assess the edge cases. For more details on this methodology, please refer to [17].

The count on each detector is depicted with the corresponding color of Figure 6a. The simulation ran at a traffic light cycle of 15 s, no offset between intersections, with a vehicle queue from the south of 6 left (CTG), 6 right (CTG), 18 straight (CTG), 8 left (CDG), 8 right (CDG), and 21 straight (CDG). Around a simulation time of 150 s, a slightly higher compactness of the CTG platoon in SUMO causes a time difference at the intermediate straight detector of 1.2 s. Around a time of 239 s, the CDG platoon of 20 vehicles stops in front of the north intersection. The 15^th^ vehicle stops right on the same detectors in PHABMACS, while in SUMO the corresponding vehicle stops slightly in front of the detector. Thus, a time difference of a full cycle time is measured. The final parameterization of the SUMO model after calibration is listed below in Table 2, encompassing notation from both this work (1) and the SUMO user documentation [25], aiming to facilitate SUMO developers to reproduce the results of this work.

### 4.3. Step II—Metric Validation

**Figure 7:**
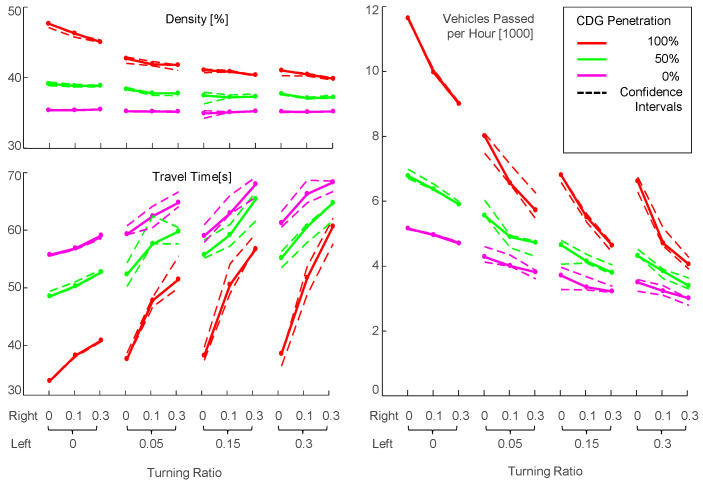
CDG/CTG model metric validation for SUMO-PHABMACS.

In step II, the calibration completed in step I is validated. The approach of our proposed validation method is based on the statistical analysis of the same simulation scenario in both simulators. By following a similar approach to the validation of a sub-microscopic simulation model against a real-world vehicle in [17], we now validate a microscopic traffic simulator (SUMO) against a sub-microscopic vehicle simulator (PHABMACS). As described in [27], we employ the 95% confidence interval of the relevant metric measured at multiple simulation repetitions for analysis. The confidence intervals for each metric (see Section 3.5) is depicted in Figure 7.

The CDG/CTG model metric validation for the 15 s phase time simulation run corresponding with [1] (see Section IV.C, Figure 9 in [1]). The confidence intervals are determined according to (2), as described in [17], using the MATLAB^®®^ implementations of the Student’s t inverse cumulative distribution function “*tinv*”, and the standard deviation “*std*” for σ, where v is the degrees of freedom (the number of simulations, six in this case) and µ is the mean value of the data.

(2) $$U,L=\left\{\mu \mp C\frac{\sigma }{\sqrt{N}}\right\},\;C=tinv\left(0.95,v\right),\;N=\frac{v}{2}-1$$

All permutations were simulated six times in PHABMACS and in SUMO. We consider validity as achieved if the average metric measured in SUMO is inside the confidence band measured in PHABMACS, which is the case, as shown in Figure 7.

## 5. Extensions to the Simulation Models of Original Study

In this section, we describe the extensions made to the simulation models of our original study, introducing the implementation of the three measures PLL, IA, and CMG, aimed at mitigating performance drops resulting from high penetration rates of CDG in multi-intersection scenarios. Their implementation is based on a decentralized platooning approach, as motivated based on Section 2.

**Remark 1.**  *As discussed in Section 2, these three measures would be most effectively implemented by introducing a formal platoon architecture, which would simplify coordination and enhance string stability. However, a fully networked platoon [28] poses the disadvantage of significant communication overhead that could overload wireless communication in densely populated traffic scenarios. Additionally, in such scenarios, correctly identifying and locating all vehicles mutually and assigning them to different platoons becomes challenging. Therefore, a key assumption in our study is to leverage the advantages of a one-vehicle-lookahead approach in these scenarios, which necessitates only identifying and receiving information from the immediately preceding vehicle. We assume that it is worth accepting potential drawbacks regarding string stability and rely on the one-vehicle-lookahead communication pattern in the best case, or in unavoidable circumstances degrade to mini-platoons [2]. This exhibits the lowest possible communication overhead compared to the one-vehicle-lookahead pattern in oversaturated multi-intersection scenarios with a high proportion of V2X-enabled vehicles*.

### 5.1. Limiting the Platoon Length

**Figure 8:**
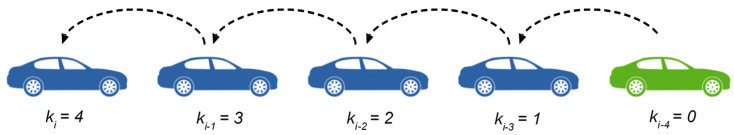
One-vehicle-lookahead communication structure with example of limiting the platoon size to four followers; ${\;k}_{tr}$ = 5.

In order to enable the limitation of the CDG platoon length, without having a formal platoon architecture, we employ the simple scheme depicted in Figure 8. Each vehicle i includes a number ${\;k}_{i}$ in the communication sent to its follower, for which (3) holds ($k_{i}$ = 0 if i has no predecessor, and ${\;k}_{i}={\;k}_{i-1}+1$ otherwise). If ${\;k}_{i}$ exceeds a threshold ${\;k}_{tr}$, which defines the limit of the platoon length, then i employs the CTG spacing policy). To enable this approach, we assume all communicating vehicles to be able to identify their direct preceding vehicle to receive information from and to have a unique identifier, e.g., via a public key infrastructure, as already operational in the EU and the US [29].

(3) $${\;k}_{i}=\left\{\begin{array}{cc} 0 & if\;i\;has\;no\;predecessor\\ 0 & if\;{\;k}_{i-1}>{\;k}_{tr}\to CTG\;is\;applied\\ {\;\;\;k}_{i-1}+1 & otherwise \end{array}\right.\;$$

### 5.2. Intersection Awareness

In scenarios where traffic congestion extends from one traffic light to an adjacent intersection, vehicles may stop within the intersection until the opposing traffic light cycle clears. In this situation, the cross traffic has to wait for a full traffic light cycle. Due to the close distances in CDG platoons, this event occurs more often than with CTG, which by its very nature creates a contraction of the platoon while stopping and thereby more space on the intersection area. In order to create spaces on the intersection, CDG would require a coordination between vehicles, such as described in [30].

For a real-world implementation of IA for CDG without a formal platoon architecture, such a strategy requires a map, which can be used by each vehicle i  separately to check locally if the platoon passes an intersection. This check can be performed by an estimation of the platoon length in front of vehicle i using ${\;k}_{i}$. If the current deceleration rate leads to a stop of vehicle i inside the intersection area, the vehicle switches to CTG and holds in front of the green traffic light. In SUMO, there is a heuristic mechanism (no-block heuristic) [16] that helps vehicles to anticipate a possible hold at a position which blocks the cross traffic. In our previous study [1], we used SUMO version 0.32, where the no-block heuristic did not work properly for sub-second simulations (our CDG model for SUMO requires a step size of 0.1 s). These issues were solved in SUMO version 1.6. Thus, in the present study, we could leverage the SUMO heuristic as an implementation of IA.

### 5.3. Creating Merging Gaps

The merging mechanism applied in this work, to counteract the performance drop of CDG resulting from prevented lane changes, is described in the following. As discussed previously, we aim to design a mechanism without a formal platoon architecture. This mechanism, deployed in each vehicle i in a platoon, needs an event that triggers opening a gap in front of i and a policy that describes the process of gap opening. For the purpose of simplification, we design this gap-opening policy as a simple switch from CDG to CTG, as the gaps arising in SUMO when CTG is applied turned out to be sufficiently large for the SUMO default lane change model [31] to allow lane changes [1]. This rationale aligns our methodology, given that we have calibrated the spacing behavior to emulate that of human drivers. The event triggering the gap opening is defined in a straightforward manner as follows. In the field of view of vehicle i driving on the ego lane, another vehicle j on either neighboring lane is detected visually signaling an intended change to the ego lane by an activated turn signal. Let ${\;tv}_{i}$ be the time j remains in the field of view of i and ${\;tv}_{tr}$ a threshold for which (4) holds.

(4) $${\;d}_{r,i}=\left\{\;\begin{array}{cc} r_{i}+h_{i} & if\;{tv}_{i}>{tv}_{tr}\\ r_{i} & otherwise \end{array}\right.\;$$

## 6. Results

In this section, we present the results of the large-scale simulations conducted to evaluate the impact of CDG on the traffic system, or more precisely on multiple mutually influencing intersections. We begin with the evaluation of the synthetic simulation scenarios described in Section 3.4.2 to analyze the crucial traffic hindrance situations caused by CDG, and the capability of the three measures PLL, IA, and CMG, presented in Section 5, to mitigate these situations. In addition to the synthetic grid scenario (see Section 3.4.2) we analyzed a synthetic corridor (see Section 3.4.2). In order to confirm the results using synthetic simulation scenarios, we further assessed the real-world performance of CDG on a heavily frequented arterial road in Berlin, Germany, as depicted in Figure 5 (see Section 3.4.2). All information pertaining to the simulation models, their parameters and assumptions beyond the information given in Section 3, needed to reproduce the results can be found in [1] and its references. The three measures described in Section 5 to create a close-range coordination between vehicles to mitigate disturbance effects were parametrized as described in Section 5, and summarized in Table 3, for all subsequent simulations. Their impact on the results is discussed in the rest of this section.

### 6.1. Synthetic Arterial Scenario

The synthetic arterial scenario represents five coordinated intersections along a major street (see Section 3.4.2). The green-light portion of the cycle time is longer for the major street than for the minor streets. For further detailed information on the considerations that led to this approach and further details on the scenario configuration, refer to [1]. The following parameters were applied for the simulation:

- maximum possible traffic inflows are specified at all inlets;
- turning rates on minor roads: left 20%, right 40%;
- turning rates on main road is permuted with 1 (no turning), 2 (left 10%, right 20%);
- penetration rates are permuted with 0% (CTG), 50% (Mix), and 100% (CDG);
- green-light portion for the major street is permuted with 25 s, 30 s, and 35 s, with corresponding 10 s, 7 s, and 5 s for the minor streets;
- offset time between intersection cycles is permuted with 0 s and 15 s.

#### 6.1.1. Arterial Scenario without Turnings on the Major Road

**Figure 9:**
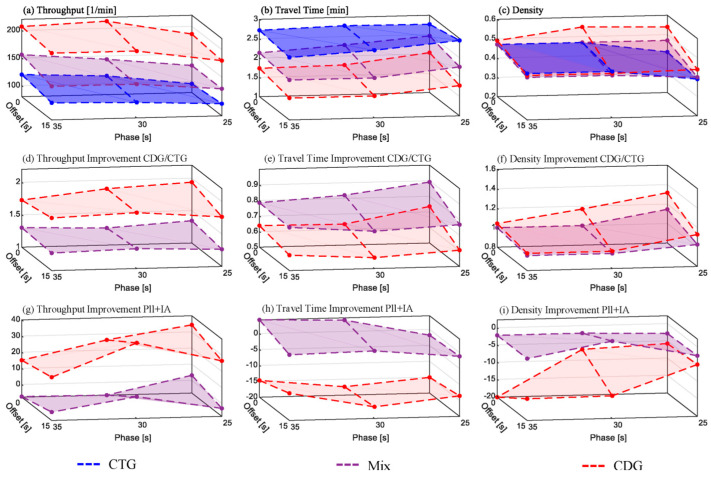
Arterial scenario simulation results without turnings. Subfigure descriptions: (**a**–**c**)—absolute values; (**d**–**f**)—improvement for CDG/CTG; (**g**–**i**)—improvement related to PLL and IA.

The results depicted in Figure 9 are related to the synthetic arterial scenario described in Section 3.4.2 with rates of 20% left turnings and 40% right turnings on the minor roads and without turnings on the major road. Figure 9a–c show the absolute values of the selected metrics throughput, travel time, and traffic density (see Section 3.5) measured for *CDG*, *CTG*, and *Mix*. Figure 9d–f depict the improvement of *CDG* and *Mix* over *CTG* as a quotient. In each subfigure, the relevant metric is plotted at the vertical axis on a ground plane which represents the permutation of green time and offset. Figure 9g–I in a similar manner, show the respective contributions of the three measures to the improvement over CTG. This contribution is calculated by the disparity between the improvement over CTG in the absence of these three measures and their inclusion.

In the original study [1], both CTG and CDG throughput increased with longer green times. A phase offset between adjacent traffic lights slightly negatively affected both above a 30 s green time. CDG showed an average improvement of around 50%, while Mix was around 35%. This overall improvement was lower than in the single isolated traffic light scenario [1] due to junction and turn blocking. PLL and IA mitigated intersection and turn blocking as expected. Without turning vehicles on the main road, both measures significantly improved CDG performance. The highest CDG throughput increase compared to CTG, at 25 s of green time without offset it reached 93%, a 35% increase from the original study (see Figure 9g). The most notable effects of PLL and IA were at 30 s of green time and 15 s offset, yielding a 39% throughput increase and 56% travel time savings for CDG, representing a 17% reduction (see Figure 9h).

IA effectively kept intersections clear, preventing junction blocking, though turn blocking still occurred due to insufficient space to allow turning from the minor streets. An alternative IA parameterization, not presented as a figure, prevented turn blocking but reduced main road throughput, negating average throughput gains.

Thus, while PLL and IA enhanced the throughput, the CDG overall improvement potential in this scenario saturates for green times exceeding 30 s on the main road.

#### 6.1.2. Arterial Scenario with Turnings on the Major Road

No significant performance improvement was achieved in the arterial scenario with additional turnings on the main street. Thus, visual presentation of the results is omitted. CDG combined with PLL and IA improved over CTG, peaking at 109% at 35 s of green time, as in the original study. The difference from the case without turnings is due to gaps left by turning vehicles, allowing platoons to contract at red lights and mitigating junction and turn blocking. CTG was negatively affected by turnings, especially at longer green times, whereas CDG reached its full potential without needing PLL and IA. The shorter platoon sizes had a slightly negative impact on performance at longer green times.

### 6.2. Synthetic Grid Scenario

The synthetic grid scenario (see Section 3.4.2) includes 25 adjacent intersections in a coordinated grid network [1] of traffic light-controlled intersections that connect major streets. Thus, the green-light portion of the cycle time is equal for both directions. The following parameters are applied for the simulation:

- maximum possible traffic inflows are specified at all inlets;
- turning rates at all intersections are permuted: 1 (no turnings), 2 (left 5%, right 10%);
- penetration rates are permuted with 0% (CTG), 50% (Mix), and 100% (CDG);
- green-light portion is permuted with 5 s, 10 s, 15 s, 20 s;
- offset time between intersections is permuted with 0 s, 5 s, 10 s, and 15 s.

#### 6.2.1. Grid Scenario without Turnings

**Figure 10:**
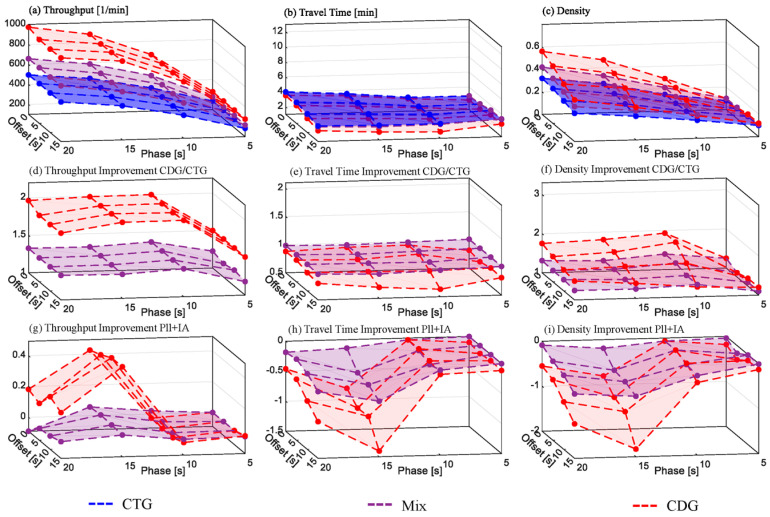
Grid scenario simulation results without turnings. Subfigure descriptions: (**a**–**c**)—absolute values; (**d**–**f**)—improvement for CDG/CTG; (**g**–**i**)—improvement related to PLL and IA.

**Figure 11:**
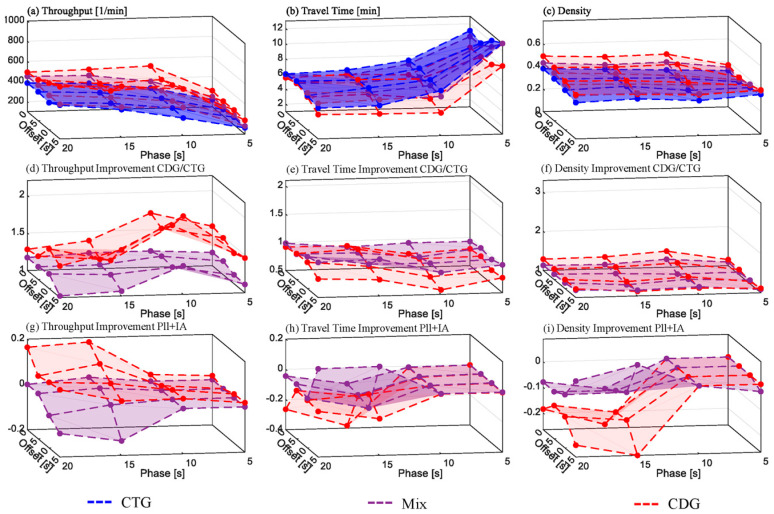
Grid scenario simulation results with turnings. Subfigure descriptions: (**a**–**c**)—absolute values; (**d**–**f**)—improvement for CDG/CTG; (**g**–**i**)—improvement related to PLL and IA.

Figure 10 depicts the grid simulation results without turnings and Figure 11 with turnings (5% left, 10% right). The subfigure structure is similar to Figure 9.

As shown in Figure 10d, the traffic throughput increase by CDG over CTG reaches approximately 100% at green-phase durations of 10 s and above. The most significant enhancement gain due to PLL and IA, over 40%, occurs at a 15 s green phase (Figure 10g). This occurs because, without platoon length limitation, longer CDG platoons exceed the intersection interspaces, potentially causing junction blocking, as observed in our original study. From 15 s and beyond, PLL and IA can fully leverage their benefits. Initially, travel time increased with CDG compared to CTG, but with PLL and IA, there is now a 104% travel time improvement over the CDG performance in the original study. The throughput improvement with PLL and IA reaches saturation at around 100% from 10 s (Figure 10g). While PLL counteracts junction blocking, it limits throughput improvement over CTG for green phases longer than 15 s, as seen in the slight decrease beyond a 20 s green phase.

#### 6.2.2. Grid Scenario with Turnings

As depicted in Figure 11, the improvement potential from PLL and IA in the grid scenario with 5% left turnings and 10% right turnings is predictably lower compared to the same scenario without turnings. The most substantial CDG throughput increase over CTG is 103% for a green phase of 10 s and a 15 s offset (Figure 11a). However, at a 15 s green phase, the improvement is much lower, reaching only 28% at a 20 s green phase. An offset contributes to throughput improvement, with a maximum of 52% observed at a 10 s offset, consistent with the original study.

For green phases up to 10 s, PLL and IA yield marginal improvements for CDG. For longer green phases, they mitigate adverse effects from the absence of offsets, leading to a 20% increase in CDG throughput (Figure 11g). This shows that without offsets CDG can achieve around 20% more throughput with PLL and IA.

The reasons for this can be explained as follows. The primary obstacle for CDG performance improvements in the grid scenario with turnings is turn blocking, particularly right turns. While junction blocking can be effectively prevented by the intersection awareness (IA) measure, mitigating turn blocking would necessitate ensuring even more space at the end of the intersection through appropriate parameterization of the IA threshold. However, at a ratio of 10% for right-turning vehicles, this space would not be sufficiently utilized by turning vehicles to compensate for the resulting reduction in throughput for straight-driving vehicles. Thus, the throughput gain would be leveled out and would not be discernible in the averaged throughput measurement.

This is further evident as the throughput for *Mix*, in some cases, is worse than without the IA because IA in these cases leaves more space than necessary at the end of the intersection. This specifically occurs in cases where IA would not be necessary due to the positive influence of the offset of green-light phases between adjacent intersections and the mixture with CTG vehicles. A similar pattern emerges in the measured travel time.

A preliminary conclusion here is that, as expected, IA only contributes to improvements at high penetration rates and would not be necessary for mixed traffic and certain offset configurations.

### 6.3. Real-World Scenario

In contrast to the simulations in the synthetic scenarios, lane changes are not neglected in the real-world road network scenario (see Section 3.4.2), in the original study [1] junction blocking and turn blocking posed minimal issues in this scenario. However, the dense traffic with small gaps at high CDG penetration rates caused challenges for vehicles performing lane changes, consequently causing traffic congestion and a decline in overall performance.

**Figure 12:**
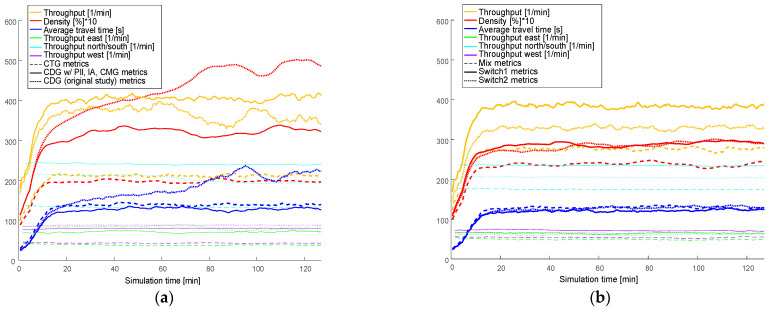
Simulation results of real-world scenario for Berlin: (**a**) Comparison of CDG combined with PLL, AI, and CMG with CTG and with the original study [1]; (**b**) comparison of the policies Mix, SWITCH1, and SWITCH2.

Figure 12 illustrates the outcome of simulations incorporating all three measures: PLL, IA, and CMG. The baseline, CTG, reaches a traffic throughput of around 210 vehicles per minute after approximately 15 min simulation time, while 100% CDG penetration reaches a throughput of around 400 vehicles per minute. This is an improvement compared to the original study, which peaked at 380 vehicles per minute before declining to 355 due to congestion, now mitigated by the new measures. The travel time for CDG is approximately 145 s, a reduction of 10% compared to CTG. For comparison, Figure 12 depicts the values for CDG from the original study. The simulation yielded similar values for Mix and SWITCH2 as in the original study. However, SWITCH1, like CDG, also benefited from the combination of the three measures PLL, IA, and CMG, particularly due to the improved lane-changing behavior, resulting in a slight throughput increase from 375 to approximately 390 vehicles per minute.

## 7. Discussion

The hypothesis of our previous work [1] has been confirmed regarding the impact of combining CDG with a close-range coordination in the form of the three measures that were the subject of investigation in this work. The results of all simulations are discussed in the following and summarized in Figure 9, Figure 10, Figure 11 and Figure 12 and Table 4 and Table 5. The percentage spans in the tables refer to the simulation runs of each scenario with the highest and lowest improvement. The contributions of IA, PLL, and CMG are calculated based on the average of all simulations, which determines the disparity between the improvement of CDG over CTG in the absence of these three measures and their inclusion.

In the arterial scenario with no turnings on the major road, the positive impact of the two measures platoon length limitation (PLL) and intersection awareness (IA) led to the expected mitigation of junction blocking and turning blocking. This resulted in an average throughput increase of 27% (15–39%) and a 15% (15–17%) travel time reduction compared to the original study utilizing CDG without these measures. While PLL counteracts junction blocking, it limits the achievable throughput improvement over CTG. At green-phase durations exceeding 15 s, the throughput improvement of CDG over CTG declines when the platoon length is limited to 20 vehicles. This occurs since a platoon of 20 vehicles need a green phase beyond 15 s to cross the traffic light at once (refer to [1], Figure 5). Consequently, while the PLL and IA enhanced the throughput improvement potential of CDG, it reaches saturation in this scenario for green-phase durations exceeding 30 s on the main road.

A similar trend emerges in the grid scenario with no turnings. Here, the throughput improvement of CDG over CTG begins to slightly decrease at 20 s green-phase lengths due to PLL. Nevertheless, the overall positive effect of the PLL and IA measures remains considerably more pronounced here than in the arterial scenario. At a 15 s green phase the throughput improvement of CDG reaches 40% and the travel time improvement is 104% compared to the original study, utilizing CDG without these measures. In the grid scenario with turning, the two measures yield less pronounced improvements for CDG. Despite successfully preventing gridlocks, IA contributed less than desired to preventing turn blocking. The negative impact on CDG performance due to the absence of offsets in the traffic light configuration could be mitigated but not entirely eliminated. Consequently, the assessment from the original study [1] remains, that a 100% CDG penetration in oversaturated grids has limited potential for throughput improvement compared to CTG. Beyond 20 s green-phase lengths, CDG can achieve only a 35% throughput improvement over CTG, provided that traffic control is not aligned accordingly. For instance, through appropriate green-phase lengths and offsets between intersections.

While in the arterial and the grid scenario, the isolated effect of PLL and IA could be validated, the positive impact of the measure creating merging gaps (CMG) became apparent in the real-world scenario as expected. The traffic congestion resulting from prevented lane changes, as observed in the original study [1], was effectively mitigated by CMG. This demonstrated the feasibility of deploying high CDG penetration rates in the real world, leading to a throughput increase of around 90% of CDG over CTG. This is a notable 20% increase compared to an application of CDG without the three measures. In this scenario, there was no noticeable reduction in throughput due to the platoon length limitation because the green-phase durations in the real-world scenario did not extend far beyond 20 s (refer to [1], Table 2). The reason why only CDG and SWITCH1 benefited from CMG is that with all other policies, the gaps between vehicles were already large enough to allow lane changes, even without CMG.

Similar to the original study, SWITCH1 demonstrates a potential comparable to CDG in real-world deployment. This suggests that at high penetration rates, opting for a policy aligned with SWITCH1 might be more advantageous than consistently relying on CDG.

## 8. Conclusions

In our previous work, we were able to show the enormous potential of CDG to increase the capacity of single-signal intersections in an oversaturated condition. However, it revealed a potential performance drop of CDG originating from prevented lane changing and blocked intersections due to missing coordination and small gaps.

In order to tackle these issues by a close-range coordination between vehicles, in this work, we introduce three measures that should be combined with CDG when employed in large and oversaturated traffic systems, platoon length limitation (PLL), intersection awareness (IA), and creating merging gaps (CMG).

These measures were designed to operate using the one-vehicle-lookahead pattern and without a formal platoon architecture, while also interacting with vehicles not equipped with communication technology. This results in a reduction in coordination complexity and reduces the wireless communication channel’s load, enabling the application of CDG in scenarios involving a large number of vehicles.

In order to enable large-scale traffic simulations of several thousand vehicles using SUMO, we propose a methodology to calibrate and validate a sub-microscopic simulation model against a microscopic simulation model. This method can be used to transfer the precision of simulation of a CACC controller in a vehicle dynamics simulation to the traffic simulation level. In this way, CDG can be evaluated regarding its impact on whole traffic systems, i.e., on multiple mutually influencing intersections.

All three measures, PLL, IA, and CMG, have largely met the expectations placed on them in our previous work. The limitations on the applicability of CDG in traffic systems, as identified in the original study, have been largely eliminated. Thus, the hypothesis of our previous work regarding the impact of the three measures has been confirmed. However, only the expectations of IA to prevent turn blocking were not entirely met, while IA was successfully employed to prevent junction blocking. For future work, this suggests the need to discover measures that can more effectively address turn blocking.

## Figures and Tables

**Table 1:** Distance policies studied in this work.

Policy	Formal Description	Parameterization
CDG	${\;d}_{r,i}=r_{i}$	$r_{i}=2.95\;m$
CTG	${\;d}_{r,i}=r_{i}+h_{i}v_{i}$	$r_{i}=2.95\;m,\;h_{i}=0.87$
Mix	${\;d}_{r,i}=\left\{\begin{array}{c} r_{i}\\ r_{i}+h_{i}v_{i} \end{array}\begin{array}{c} \;\;\;w/\;probability\;0.5\\ \;\;\;w/\;probability\;0.5 \end{array}\right.$	$r_{i}=2.95\;m,\;h_{i}=0.87$
SWITCH1	${\;d}_{r,i}=r_{i}+h_{i}{max(0,v}_{i}{-v}_{lim})$	$r_{i}=2.95\;m,\;h_{d,i}=0.87,v_{lim}=30\;km/h$
SWITCH2	${\;d}_{r,i}=r_{i}+h_{i}{max(0,v}_{i}{-v}_{lim})$	$r_{i}=2.95\;m,\;h_{d,i}=2.17,v_{lim}=30\;km/h$

**Table 2:** Final SUMO model parameters resulting from calibration and validation.

Model	Parameter in SUMO				
	decel (b)	accel (a)	tau (τ)	minGap (s_0_)	Sigma
CTH	4.70	1.70	0.9	2.95	0.4
CDG	4.70	1.40	0.02	2.45 (+s_t_ 0.5)	0.02

**Table 3:** Parameterization of the three measures PLL, IA, and CMG.

Measure	Parameterization
Platoon Length Limitation (PLL)	${\;k}_{tr}$ = 20
Intersection Awareness (IA)	SUMO no-block-heuristic [16]
Creating Merging Gaps (CMG)	${tv}_{tr}$ = 10

**Table 4:** Increase in throughput achieved by CDG compared to CTG in each respective scenario.

Scenario	Throughput Improvement CTG vs. CDG w/ IA, PLL, and CMG	Contribution of IA, PLL, and CMG
Synthetic arterial w/o turnings	73–93%	27% (15–39%)
Synthetic arterial w/ turnings	51–109%	0% (0–0%)
Synthetic grid scenario w/o turnings	50–100%	16% (−3–50%)
Synthetic grid scenario w/ turnings	28–104%	7% (0–18%)
Real-world scenario	90% (400 v/h)	20%

**Table 5:** Reduction in travel time achieved by CDG compared to CTG in each respective scenario.

Scenario	Travel Time Reduction CTG vs. CDG w/ IA, PLL, and CMG	Contribution of IA, PLL, and CMG
Synthetic arterial w/o turnings	27–43%	15% (15–17%)
Synthetic arterial w/ turnings	14–44%	0% (0–0%)
Grid scenario w/o turnings	8–38%	47% ^1^ (3–140%)
Grid scenario w/ turnings	9–42%	8% (0–38%)
Real-world scenario	10%	70%

^1^ A percentage contribution of IA, PLL, and CMG that exceeds the overall improvement signifies that without these three measures CDG would have shown a deterioration compared to CTG.

## Data Availability

The original contributions presented in the study are included in the article, further inquiries can be directed to the corresponding author/s.

## Abbreviations

**Term**	**Description**
CTG	Constant time gap—vehicle interspacing increases linearly with speed
CDG	Constant distance gap—vehicle interspacing with a fixed (usually small) distance
CACC	Cooperative adaptive cruise control—communication-enabled vehicle following
PLL	Platoon length limitation—mechanisms to control the lengths of platoons
IA	Intersection awareness—mechanisms to preventing vehicles from entering intersections when stopping within the intersection area is likely
CMG	Creating merging gaps—mechanisms to create gaps within platoons to allow for merging and lane changes
SUMO	A traffic simulator that uses microscopic simulation models
PHABMACS	A vehicle simulator that uses sub-microscopic simulation models
SWITCH	Vehicle interspacing defined by a context-aware switch between CTG and CDG
Traffic throughput	Metric defined by the number of vehicles passing per time [1/min]
Travel time [min]	Metric defined by average time vehicles need to pass [min]
Traffic density [%]	Portion of road meters occupied by vehicles [%]
V2X	Vehicle-2-X—communication among vehicles and infrastructure
Platoon	Multiple automated vehicles driving in a queue
Synthetic simulation scenarios	Scenarios for isolating and analyzing the impact of specific variables
Real-world simulation scenarios	Scenarios replicating real-world conditions to provide a realistic assessment
Multi-intersection scenarios	Scenarios of multiple interconnected and mutually influencing intersections to study the combined effects of their interactions on traffic flow
One-vehicle lookahead	Communication pattern for platoons where each vehicle receives data only from its direct preceding vehicle
Junction blocking	Traffic backlog from a traffic light reaches the adjacent intersection causing the cross traffic to have to wait for a full traffic light cycle until the intersection is clear
Turn blocking	Traffic backlogs preventing the vehicles of the cross traffic from turning
Grid lock	Traffic situation where all the roads in a particular area become so congested with vehicles that traffic comes to a complete standstill
